# FAP promotes clear cell renal cell carcinoma progression via activating the PI3K/AKT/mTOR signaling pathway

**DOI:** 10.1186/s12935-023-03073-8

**Published:** 2023-09-27

**Authors:** Kun Jiang, Li-zhe Xu, Jin-zhuo Ning, Fan Cheng

**Affiliations:** Department of Urology, Renmin Hospital of Wuhan University, Wuhan, 430060 Hubei Province People’s Republic of China

**Keywords:** Clear cell renal cell carcinoma, FAP, Prognostic biomarker, Oncogene, PI3K/AKT/mTOR signaling

## Abstract

**Objective:**

Herein, we aimed at exploring the FAP expression in clear cell renal cell carcinoma (ccRCC) along with its clinical implication.

**Methods:**

Using computational tools analysis of different freely accessible gene databases, the expression pattern, clinical importance, co-expressed genes, and signaling pathways of FAP in ccRCC were thoroughly investigated. FAP expression was examined in clinical ccRCC specimens through qRT-PCR, western blotting and immunohistochemistry. Furthermore, in vitro and in vivo experiments were carried out using flow cytometry, CCK-8, wound-healing and Transwell assays, as well as xenograft tumor model, respectively.

**Results:**

FAP levels were found to be significantly elevated in ccRCC based on bioinformatic data from public databases. Patients who exhibited higher expression levels of FAP had poorer prognoses, according to Kaplan–Meier analysis of survival data. In addition, diagnostic and prognostic value of FAP in ccRCC was figured out by ROC curve and prognostic nomogram model. In vitro study revealed that the over-expression FAP accelerated cell proliferation, migration as well as invasion, and suppressed cell apoptosis, but silencing of FAP had the opposite effect. FAP suppression reduced the PI3K/AKT/mTOR pathway's stimulation, whereas FAP up-regulation increased the stimulation of the pathway. Blocking the PI3K/AKT/mTOR signaling pathway with the dual PI3K/mTOR inhibitor BEZ235 repressesed cancer-promoting effect of FAP. Additionally, we found that the downregulation of FAP was effective at slowing tumor progression in vivo.

**Conclusion:**

It is possible that FAP could be a reliable biomarker for the diagnosis and prognosis of ccRCC because of its role in the ccRCC progression via triggering the PI3K/AKT/mTOR signaling pathway.

## Introduction

Renal cell carcinoma (RCC) is a common genitourinary tumor, representing approximately 5% of recently diagnosed malignancies in men and 3% in women [1]. Clear cell renal cell carcinoma (ccRCC), also known as kidney renal clear cell carcinoma (KIRC), is the most prevalent subtype of RCC, causing upwards of 175,000 deaths annually [2]. Because of its phenotypic variability and intratumoural heterogeneity, ccRCC is one of the most challenging human neoplasms [3]. The ccRCC subtype is histologically characterized by the presence of transparent round-shaped cells. Multiple cancer driver events have been identified through molecular characterization of ccRCC, including mutations and methylation differences in genes such as VHL, PBRM1, BAP1, and SETD2, as well as alterations in chromosomal structure such as loss of 3p and gain of 5q chromosomes [4]. Although multimodality treatments including surgery, chemotherapy, immunotherapy, and targeted therapy have developed, the prognosis of ccRCC patients is still poor due to the high rates of metastasis, recurrence, and drug resistance [5, 6]. The molecular basis behind ccRCC development must be investigated to develop potential therapeutic strategies.

Fibroblast activation protein alpha (FAP) is a homodimeric membrane-bound serine protease with a 95-kDa molecular weight [7, 8]. Cancer-associated fibroblasts (CAFs) are common in tumor microenvironments [9]. Crosstalk contact between CAFs and tumor cells promotes pro-invasive features, according to in vitro studies on renal cancer cell lines [10]. FAP is a significant surface marker of CAFs and closely relevant to occurrence and development of cancers [11]. Furthermore, FAP is predominantly expressed on the surface of activated fibroblasts in epithelial tumor cells and has a crucial function in governing the biology of cancerous cells, but it is infrequently expressed in healthy tissues [12]. FAP overexpression is related with higher tumors grade, aggressiveness, metastases, as well as poorer prognoses in individuals with gastric cancer [13]. Furthermore, FAP promotes gastrointestinal tumor development by boosting Wnt/catenin-dependent epithelial-mesenchymal transitions [14]. Furthermore, overexpression of FAP could promote tumorigenesis by upregulating PI3K/AKT in lung carcinoma [15]. It's still not clear how FAP contributes to the pathogenesis of ccRCC.

Hence, this research study evaluated the impacts of FAP on ccRCC and to identify the possible mechanisms by which these effects may have been achieved.

## Materials and methods

### Data source and processing

The Cancer Genome Atlas (TCGA) database (https://portal.gdc.cancer.gov/) was queried for normalized RNA-seq data and accompanying clinical and pathological characteristics for 539 ccRCC tissues as well as 72 normal tissues. The data were obtained as level 3 HTSeq fragments per kilobase per million bases (FPKM).

### TIMER database analysis

Immune cells infiltrating tumors as well as gene expression patterns in various cancers can be analyzed using the Tumor Immune Estimate Resource [16] (TIMER, https://cistrome.shinyapp.io/timer). The expression levels of FAP in different cancer types were analyzed using the TIMER database.

### Clinical significance of FAP expression in ccRCC

ROC analysis was used to evaluate the levels of FAP expression in ccRCC tumors and neighboring normal tissues in order to determine whether or not the protein's presence is a reliable indicator of ccRCC diagnoses. R's pROC package was used to construct the diagnostic ROC curve. Overall survival (OS), disease-specific survival (DSS), as well as progression-free interval (PFI) were examined in patients with ccRCC using the Kaplan–Meier (K-M) survival chart, which was used to divide the specimens into two groups based on median expression levels (high vs. low). A K-M plot was created and a log-rank test was carried out using the survival package in R. The rms R software was used to create a nomogram that contained significant clinical characteristics and a calibration plot for predicting OS in ccRCC patients. Nomogram-predicted probabilities were mapped against observed rates, and the 45° line reflected the most accurate predictions. Using a concordance index (C-index), it was determined that the nomogram was discriminating.

### UALCAN database analysis

There is a website called UALCAN [17] that gives comprehensive and interactive analyses of transcriptome data from the TCGA (http://ualcan.path.uab.edu/). In ccRCC, UALCAN was employed to examine the relationship between FAP expression levels and important clinical characteristics as nodal metastases as well as cancer subtypes.

### Functional enrichment analysis

Using the R function cor.test, the Spearman correlation coefficient was calculated for the correlation measurements. Genes were chosen based on their high positive or negative correlation coefficients with FAP. clusterProfiler was used to conduct Gene Ontology (GO), Kyoto Encyclopedia of Genes and Genomes (KEGG), and Gene Set Enrichment Analysis (GSEA). Signature sets from the Molecular Signature Database were used to conduct GSEA.

### Clinical tissues

During 2019–2021, the Ethics Committee at Renmin Hospital of Wuhan University accepted the gathering of 35 ccRCC specimens as well as normal tissues that matched them. Consents were obtained from all included patients for using their specimens and clinical data in this investigation. All specimens were separated into two groups, with half fixed in 4% paraformaldehyde and the remaining half frozen and stored at − 80 °C until analysis.

### Cell culture and transfection

Invitrogen-supplemented RPMI-1640 media (Invitrogen, USA) with 10% FBS was used to grow the human ccRCC cell lines (Caki-1, ACHN, as well as A498), as well as the normal tubular cell line HK-2. Ribo Co., Ltd. provided the FAP small interfering RNA (si-FAP), the pcDNA 3.1 FAP upregulation plasmid vector, as well as the negative controls (Wuhan, China). The Caki-1 and ACHN cell lines (1 × 10^5^) were cultured in 6-well plates until they reached 60% confluence, and then transfected with either si-FAP or FAP upregulation plasmid vector using Lipofectamine 3000 (Invitrogen, USA).

### PI3K/AKT/mTOR pathway inhibition

ACHN cells were treated with BEZ235 (a dual PI3K/mTOR inhibitor). BEZ235 was purchased from MedChemExpress (HY-50673, USA). BEZ235 was diluted in DMSO to a final concentration of 10 mM, according to the manufacturer’s instruction.

### Quantitative real-time PCR

Utilizing the TRIzol reagent, total RNA was extracted from ccRCC cells or clinical specimens (Invitrogen, USA). Utilizing the HiScript II Reverse Transcriptase Kit, RNA levels were normalized and transformed to cDNA (Vazyme, China). On a Bio-Rad system, qRT-PCR was conducted by using a a ChamQ SYBR qPCR Master Mix (Vazyme, China). GAPDH was utilized as a reference gene for the normalization of mRNA. The primer sequences are listed in Table 1.

**Table 1:** Primer sequences for qRT-PCR analysis

Target	ID Primer sequence, 5′-3′
FAP	F: CCAAAGACCCAGGAGCATATAG
	R: GTTTGTAGCCATCCTTGTCAC
GAPDH	F: CCTGCACCACCAACTGCTTA
	R: TCTTCTGGGTGGCAGTGATG

### Western blotting

Total protein was extracted utilizing RIPA buffer (Beyotime, China), then the concentration was detected utilizing a BCA protein assay kit (Beyotime, China). The proteins were separated on 10% SDS polyacrylamide gels (Solarbio, China) and afterwards transferred to PVDF membranes (Bio-Rad, USA). Following the blocking with 5% nonfat milk, incubation of blots was done overnight at 4 °C with primary antibodies against FAP (ab207178), PI3K (ab191606), p-PI3K (ab182651), AKT (ab179463), p-AKT (ab192623), mTOR (ab134903), p-mTOR (ab137133) and GADPH (ab181602), which were purchased from Abcam (Cambridge, UK). Enhanced chemiluminescence (ECL) was used to determine the signals when the secondary antibody was incubated.

### Immunohistochemistry staining

4% PFA-fixed human ccRCC tissues were dried and incorporated in paraffin before being cut into 4-μm slices. Anti-FAP antibody (Abcam, UK; ab207178) was then applied to the slides and incubated overnight at 4 °C. We next used secondary antibodies to inoculate the sections for 30 min at room temperature following they had been washed with PBS. Then after, the cells were incubated for an additional 20 min at 37 °C. with streptavidin–horseradish peroxidase (SA-HRP). Hematoxylin and bluing reagents were then used to counterstink the slides with 3, 3-diaminobenzidine tetrahydrochloride (DAB). Using an Olympus BX50 light microscope, the slides were examined (Olympus, Japan).

### Cell apoptosis assay

ACHN and Caki-1 cells (1×10 [5]) were grown in six-well plates until the cells reached 50% confluence. The apoptotic activity was detected by flow cytometry, using The Annexin V-FITC kit (Beyotime, China). The measurements of apoptotic cells were performed using FACSCanto II flow cytometer (BD, USA).

### Cell proliferation assay

Cell viability was determined using a cell counting kit-8 (CCK-8) assay. In 96-well plates, 2 × 10^3^ cells were cultivated. Every well was filled with ten microliters of CCK-8 solution prior to analysis. To quantify the absorbance of each well, the microplate reader was used following incubation for two hours (Perkin-Elmer).

### Wound healing assay

The Caki-1 and ACHN cells (5 × 10^5^) were seeded in six-well plates and incubated at 37 °C until they reached 80–90% confluence. Wounded gaps over the adherent cells were scratched by 100 µL pipette tips. Cell migration path was tracked using a microscope (Olympus, Japan) at 0 h and 24 h after scratching.

### Cell migration and invasion assays

Caki-1 and ACHN cells’ migration as well as invasion abilities may be measured utilizing Transwell chamber-based assays (Corning, USA). In the migration assay, a suspension of 1 × 10^5^ ccRCC cells in serum-free medium was introduced into the upper chambers. The lower chambers were filled with 600 μL of complete medium and subsequently incubated at 37 °C in a humidified atmosphere containing 5% CO_2_ for a duration of 24 h. After incubation, the cells that had migrated to the lower surface of the membrane were fixed using 4% paraformaldehyde and subsequently stained with crystal violet. Non-migrated cells were removed from the upper membrane surface. The migration of cells was observed and quantified using an optical microscope equipped with a 200 × magnification lens. Eight randomly selected regions were analyzed to determine the number of migrated cells. For the invasion assay, the Transwell polycarbonate membrane was coated with 5 μg of Matrigel Basement Membrane Matrix (Corning, USA) to mimic the extracellular matrix. The subsequent steps followed an identical protocol as described for the Transwell migration assay.

### Xenograft tumor model

The Animal Experiment Center at Wuhan University provided us with four-week-old female BALB/c nude mice. There were two subgroups of three mice each, who were housed in the same normal environment and had diet and water. Caki-1 cells (2 × 10^6^ cells) transfected with FAP siRNA or FAP NC were mixed with equal volume of Matrigel (BD Biosciences) and injected into the right scapular area of nude mice. Every three days after injection, the tumor volumes were measured and calculated using the formula (Length × Width^2^)/2. Tumors were harvested after 28 days of cell transfection, and the tumors weight were immediately quantified. The tumors were then collected for further investigation after the approval of the Animal Care and Use Committee of our university.

### Statistical analysis

SPSS 22.0 was used for analyzing and presenting all of the datasets as means ± standard deviations. R version 3.6.3 was used for bioinformatics analyses. In order to discover differences across cells, tissues, as well as mice, we used either the Student’s t-test or one-way analysis of variance (ANOVA). Statistical significance was defined as P < 0.05.

## Results

### Expression of FAP in ccRCC along with prognostic and diagnostic value for ccRCC

**Fig. 1:**
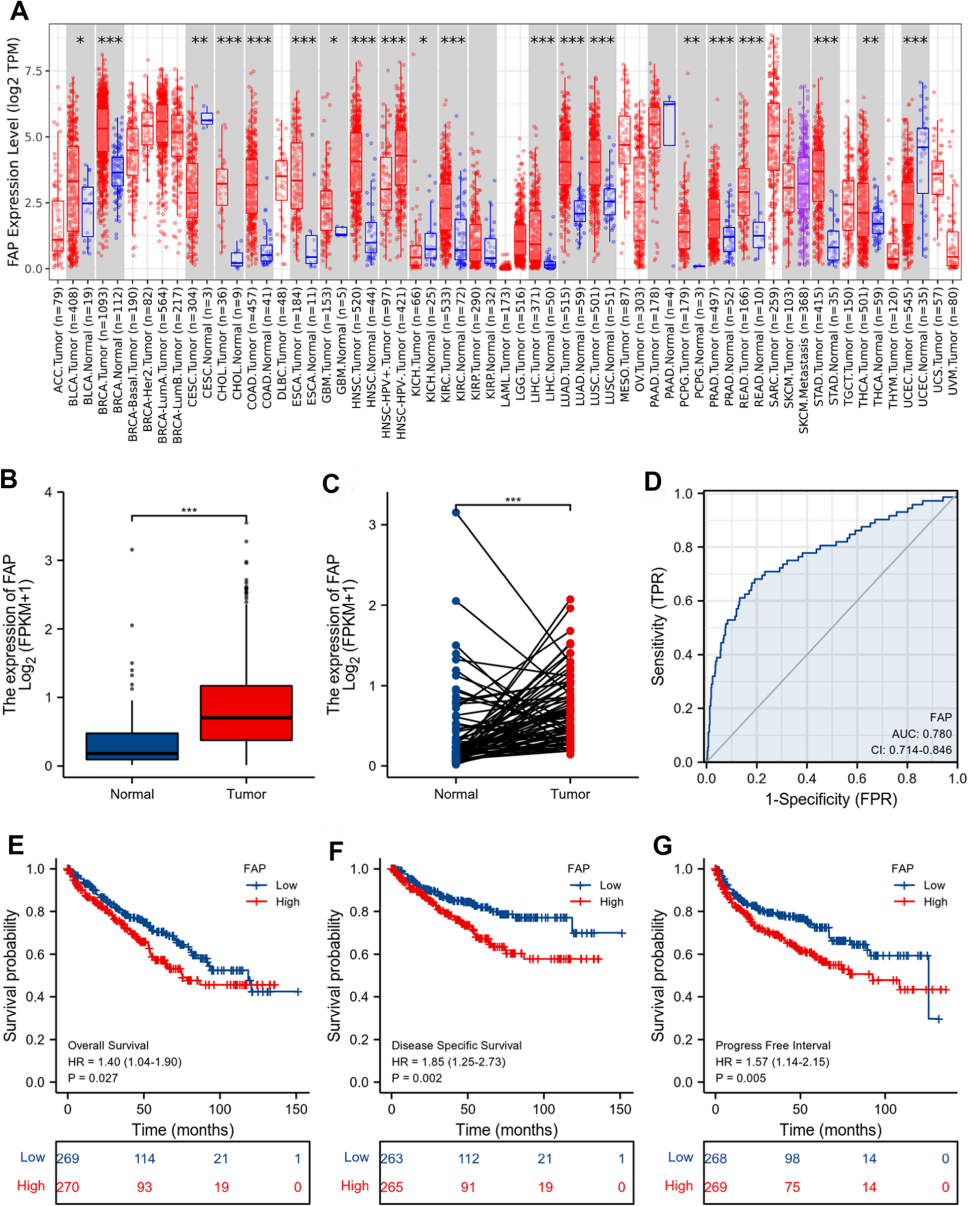
The expression of FAP is shown to be significantly increased in ccRCC, and its high expression predicts a poor prognosis. **A** FAP expression in multiple cancers based on the TIMER database. **B** In the TCGA-KIRC dataset, FAP expression was shown to be significantly higher in ccRCC tumor tissues than in normal tissues. **C** It has been shown that FAP expression is elevated in cancerous tissue relative to surrounding healthy tissues. **D** ROC analysis illustrated that AUC of 0.780 (95% confidence interval = 0.714–0.846) from the TCGA-KIRC dataset for FAP expression, which precisely distinguishes ccRCC cancer tissues from normal tissues. **E**–**G** To ensure that the results from the TCGA-KIRC dataset were statistically significant, OS, DSS, as well as PFI survival curves for ccRCC patients with high (red) and low (blue) FAP expression were all plotted. *p < 0.05; **p < 0.01; ***p < 0.001

**Fig. 2:**
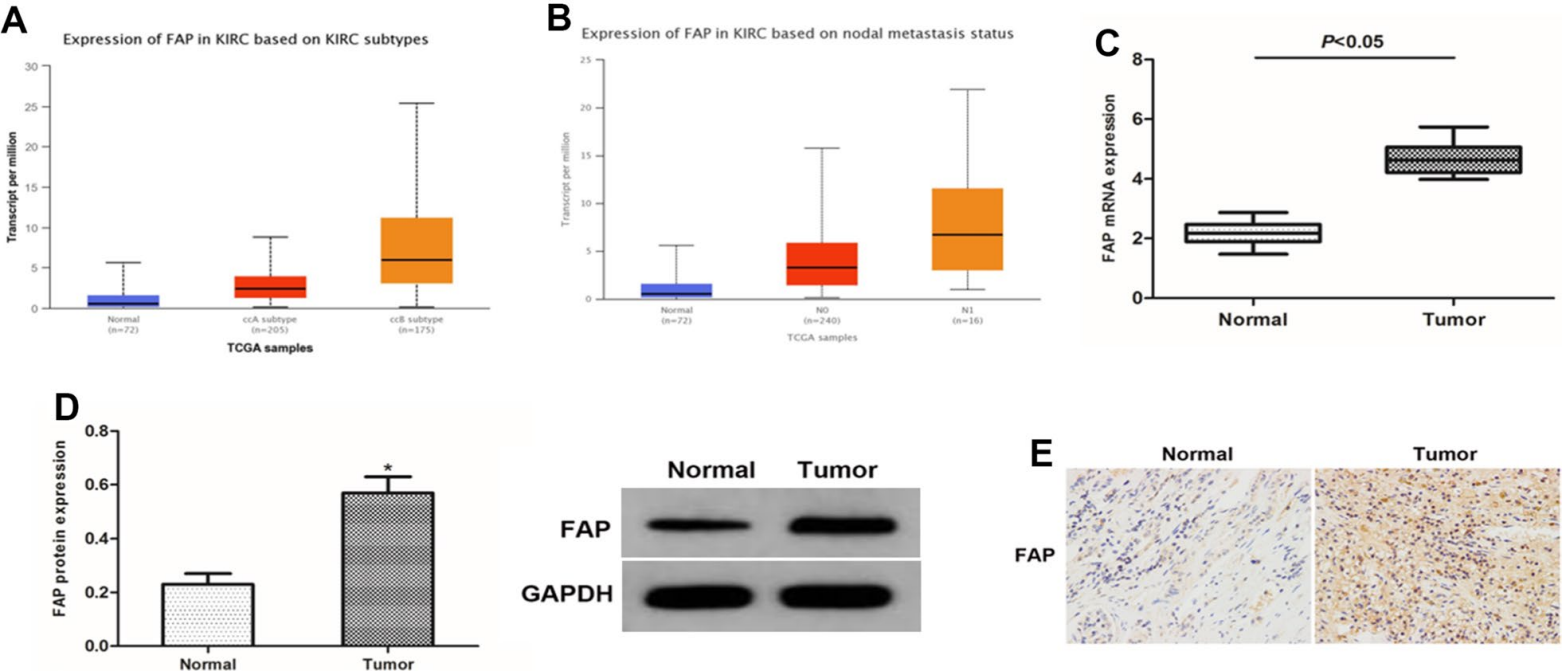
FAP mRNA and protein expression in ccRCC. **A**, **B** FAP differential expression in patient subgroups is compared to healthy controls in the UALCAN database. **C**, **D** Examination of ccRCC specimens and associated nearby normal tissues by QRT–PCR as well as western blotting **E** Tumor tissues and surrounding normal tissue samples were subjected to immunohistochemistry. * p < 0.05 vs. the Ctrl group

Using the TIMER database, we determined the FAP differential expression. which showed that FAP was significantly overexpressed in multiple cancers compared to matched control tissues (Fig. 1A). Employing TCGA data, we observed that FAP levels in ccRCC tissues were much higher than in surrounding healthy tissues (Fig. 1B, C). The prognostic reliability of FAP for ccRCC was determined using ROC analysis, which indicated AUC to be 0.780 (95% CI 0.714–0.846) (Fig. 1D). K-M curves showed that ccRCC individuals with elevated FAP expression had poorer OS (Fig. 1E), DSS (Fig. 1F), and PFI (Fig. 1G) compared to those with lower FAP expression. The connection between elevated FAP transcription and increased nodal metastatic condition as well as histological subtypes was shown to be statistically significant (Fig. 2A, B). Consequently, we analyzed the FAP expression in ccRCC samples. QRT-PCR as well as western blotting demonstrated that the concentrations of FAP transcripts and proteins were much higher in tumor tissues than in their adjacent healthy tissues (Fig. 2C, D). A similar tendency was observed with immunohistochemistry (Fig. 2E). These findings suggest that FAP expression was elevated in ccRCC tissues and linked with the development of cancer in ccRCC.

### Constructed a nomogram diagram

We established a nomogram using FAP and independent clinical risk indicators (T/N/M stage and age) to give a quantitative strategy for predicting the prognosis of ccRCC patient populations. The point was read out by drawing an upward line from each variable to the point axis for each predictive factor in the nomogram.

**Fig. 3:**
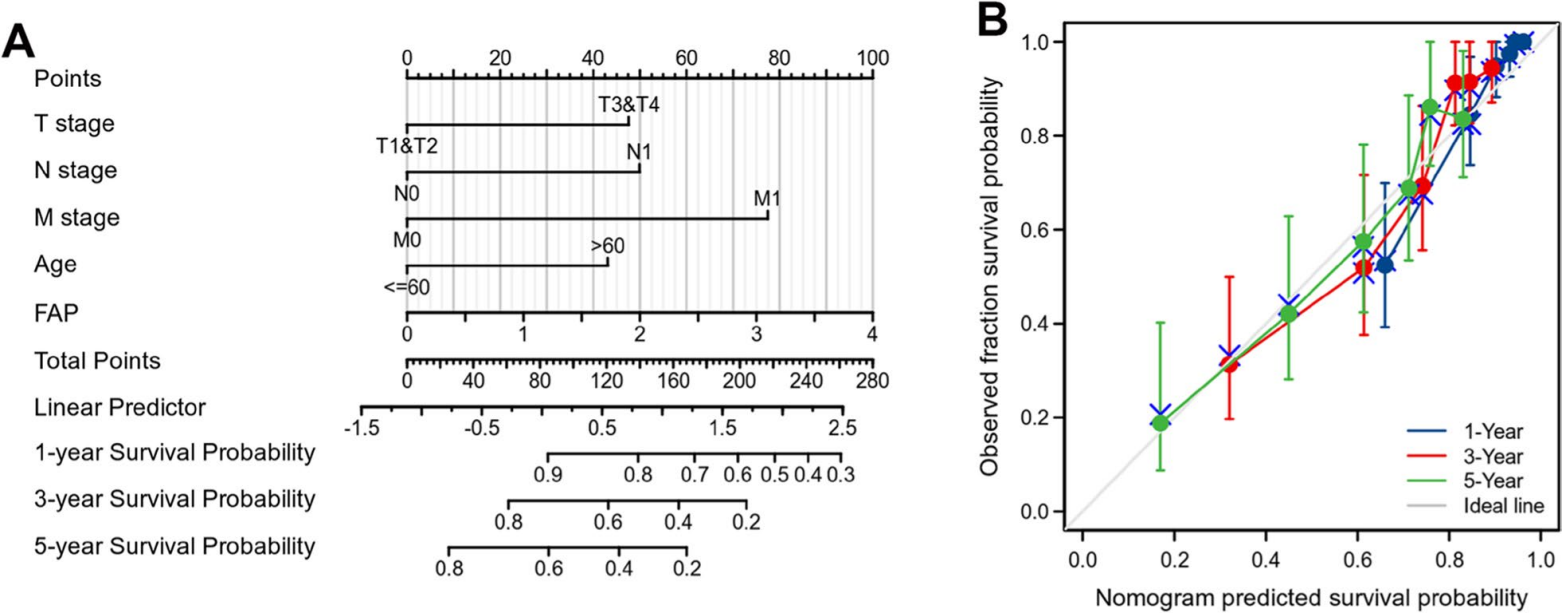
The role of FAP in predicting the prognosis of ccRCC patients. **A** FAP and independent clinical risk indicators are used in a nomogram to estimate survival probabilities for ccRCC. **B** Calibration plots of survival probabilities. The gray line signifies the actual survival

Factors were assigned a weighted number of points, which reflected a survival prognosis, for each individual. The overall points acquired by summing together the points of each element was used to predict the survival probability of 1, 3, as well as 5 years for ccRCC patients (Fig. 3A). As shown by the calibration curve, anticipated survival probabilities were in good agreement with reported survival probabilities (Fig. 3B). The OS nomogram had a C-index of 0.784 (0.761–0.808), which indicated that the model could reliably detect OS.

### FAP inhibits apoptosis and promotes proliferation of ccRCC in vitro

**Fig. 4:**
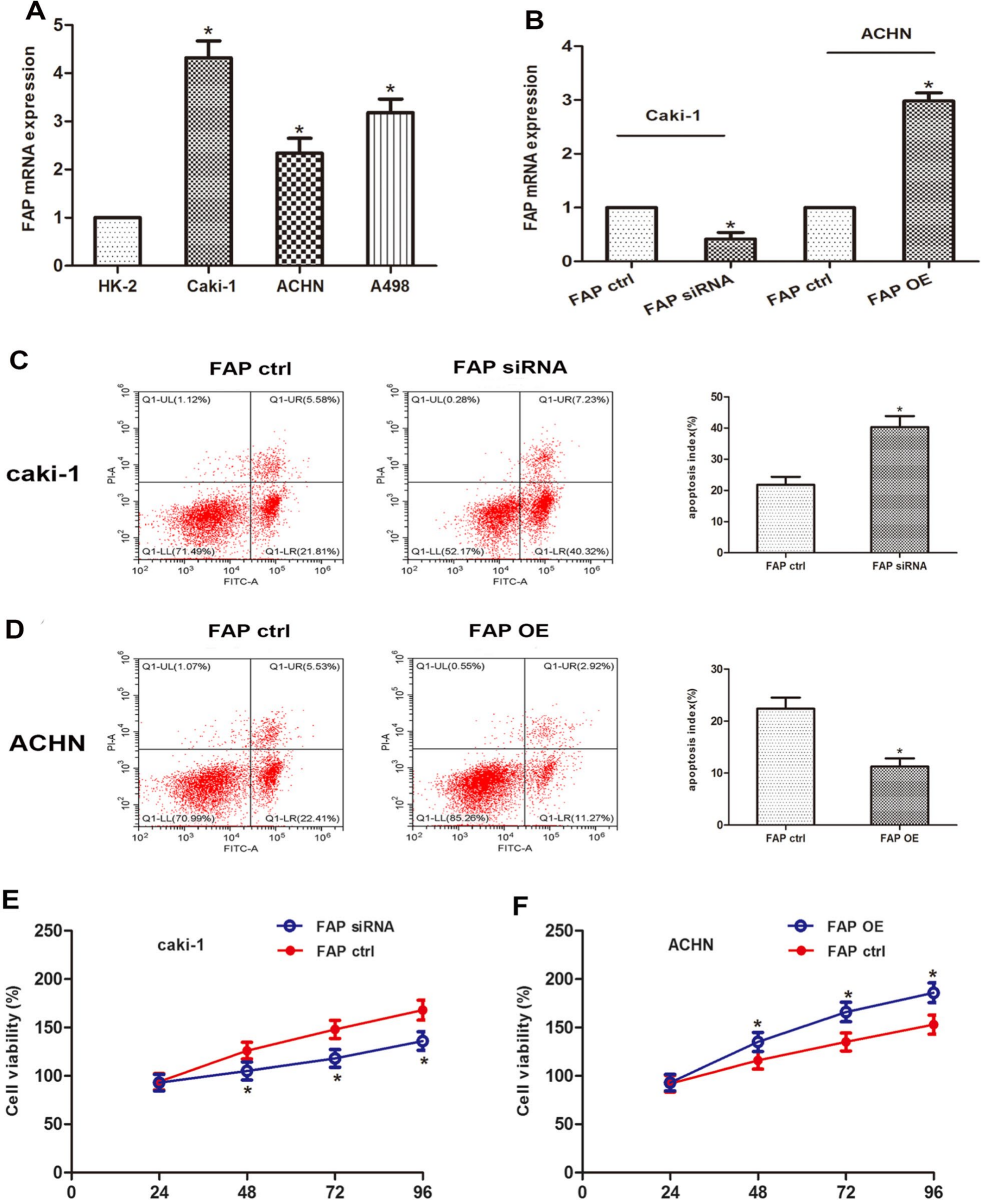
FAP suppresses apoptosis and enhances ccRCC progression. **A** FAP content in the ccRCC cell lines (Caki-1, ACHN, A498) and normal cell line (HK-2). **B** QRT-PCR was used for quantifying FAP expression levels in Caki-1 cells and ACHN cells upon transfection. **C**, **D** Flow cytometry was used to measure Caki-1 as well as ACHN cell apoptotic activities post-transfection. **E**,** F** After transfection, the CCK-8 assays were performed to detect cellular viability in Caki-1 and ACHN cell lines at 24, 48, 72, and 96 h. * p < 0.05 vs. the Ctrl group

The contents of FAP in three ccRCC cell lines (Caki-1, ACHN and A498) and non-malignant tubular cell line HK-2 was determined. The qRT-PCR showed that FAP contents were up-regulated to varying degrees in ccRCC cells (Fig. 4A). To validate the functional role of FAP in ccRCC, a si-RNA targeting FAP was transfected into Caki-1 cells and a FAP-overexpression plasmid was transfected into ACHN cells (Fig. 4B). In the next step, flow cytometry was used to measure the apoptotic frequency. When FAP expression is increased, the number of apoptotic nuclei decreases, which can be restored by lowering FAP expression (Fig. 4C, D). To further investigate the involvement of FAP in ccRCC cell proliferation, CCK-8 experiments were carried out. Cellular viability was lower in the si-FAP group compared to the NC group, but it was higher in the OE-FAP group (Fig. 4E, F). These findings imply that FAP may prevent ccRCC cells from undergoing apoptotic cell death and instead enhance their proliferation.

### FAP promotes ccRCC invasion and metastasis in vitro

**Fig. 5:**
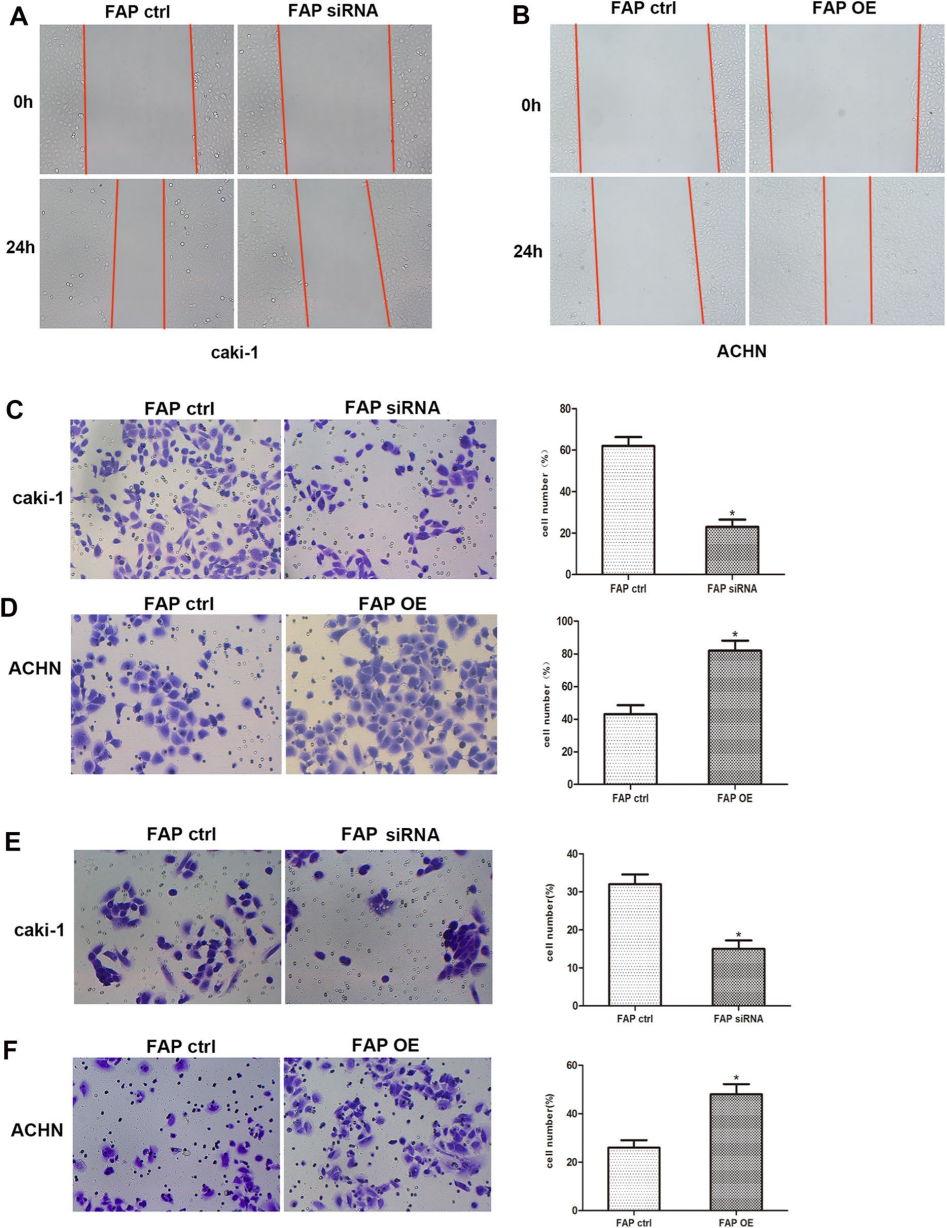
FAP stimulates ccRCC cellular migratory capacity as well as invasion in vitro. **A**, **B** Wound-healing tests were conducted 24 h following transfection to assess the migration patterns of Caki-1 as well as ACHN cells. **C**, **D** After transfection, Caki-1 as well as ACHN cells were tested for transwell migration. **E**, **F** Transwell invasion tests with Matrigel-coated membranes were conducted to determine the invasion ability of Caki-1 as well as ACHN cells following transfection. *p < 0.05 vs. the Ctrl group

We then tested the influence of FAP on the capacity of ccRCC cells to migrate and invade. The migration of ccRCC cell was significantly reduced in the wound healing experiment and the Transwell migration assay when FAP was knocked down, while FAP over-expression significantly increased ccRCC cell migration (Fig. 5A–D). The Transwell invasion assay confirmed that inhibition of FAP suppressed cell invasion, while up-regulating FAP promoted cell invasion (Fig. 5E, F). Collectively, these results suggested that FAP aggravated the aggressiveness of ccRCC.

### FAP functions via regulating the PI3K/AKT/mTOR signaling pathway

**Fig. 6:**
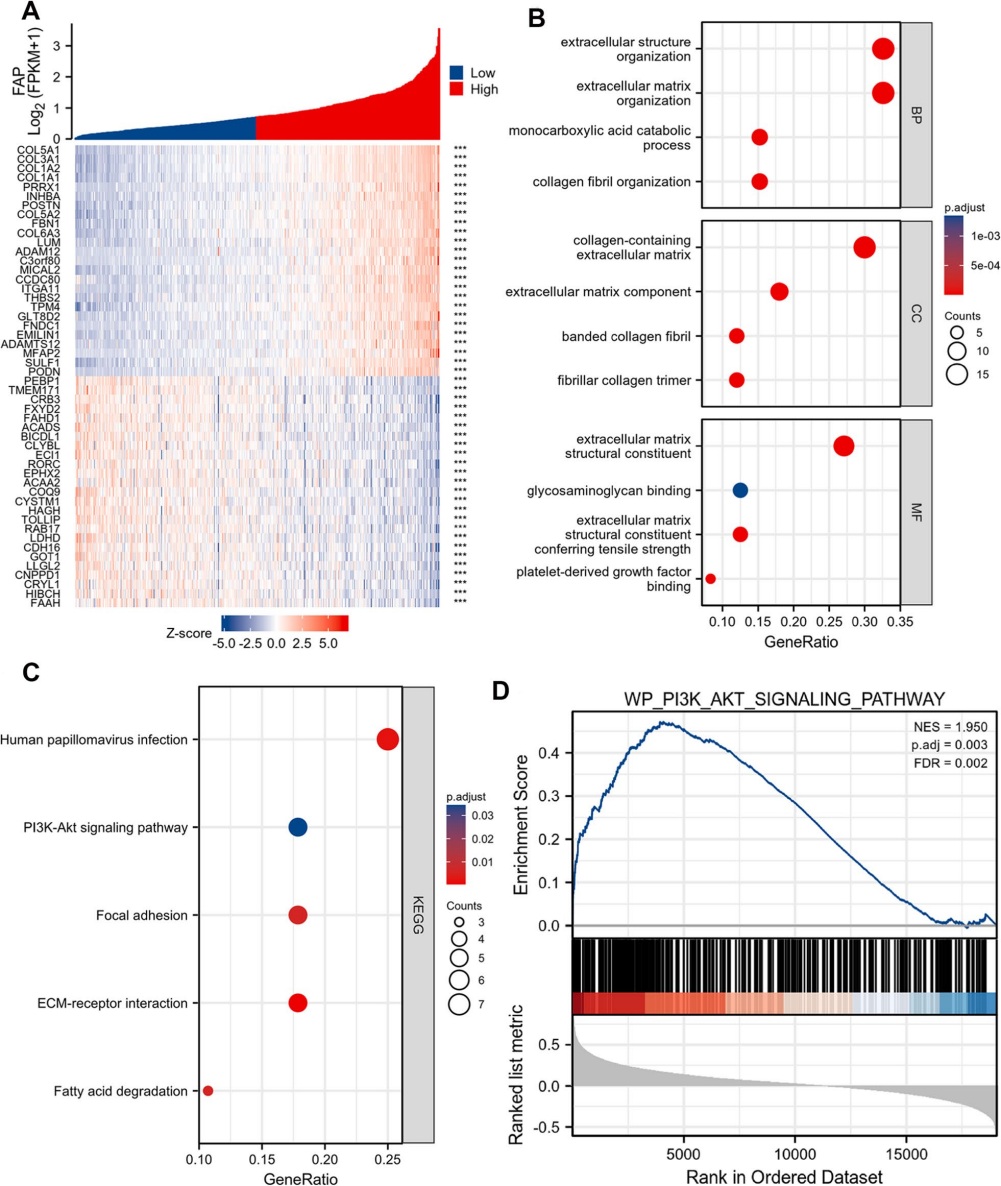
FAP co-expressed genes and functional enrichment analysis in TCGA-KIRC patients. **A** Heatmap of top 25 positively and top 25 negatively correlated genes with FAP in ccRCC. Regarding the genetic relationships, the color red symbolizes genes that are associated positively, while the color blue shows genes that are related negatively. **B**, **C** GO and KEGG pathway analyses of top 50 FAP co-expressed genes in ccRCC. **D** GSEA provided a correlation between FAP expression and PI3K/AKT pathway activation. ***p < 0.001

**Fig. 7:**
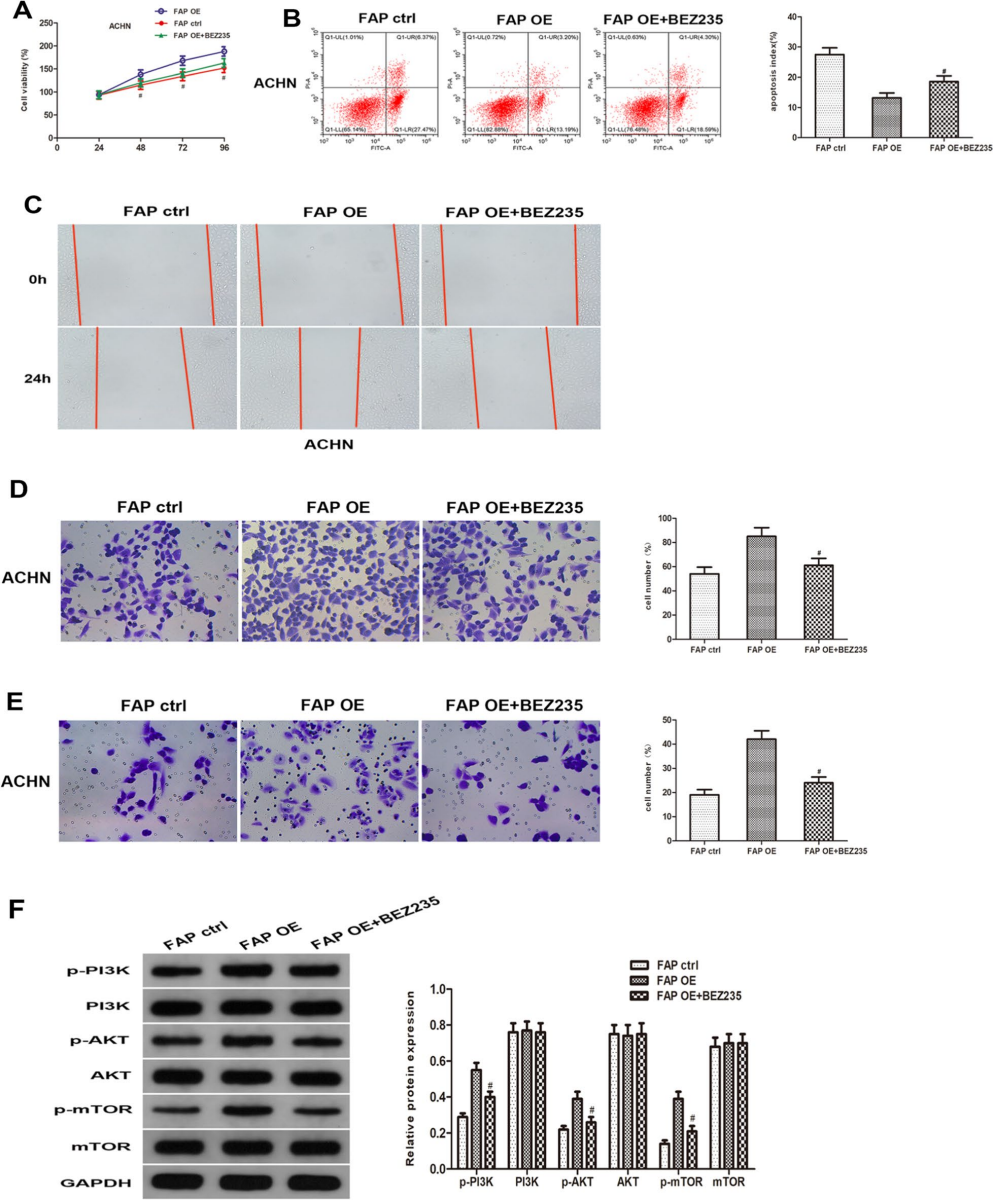
FAP activates the PI3K/AKT/mTOR pathway in vitro. **A**–**E** BEZ235, a dual PI3K/mTOR inhibitor, attenuated the FAP overexpression-induced proliferation, migration and invasion of ccRCC cells, as evidenced by the CCK-8 assays **A**, flow cytometry assays **B**, wound-healing assays **C**, transwell migration assays **D** and transwell invasion assays **E**. **F** The increases in the protein levels of p-PI3K, p-AKT, and p-mTOR after FAP overexpression were markedly attenuated by BEZ235 treatment. ^#^p < 0.05 vs. the FAP OE group

We use the TCGA database to identify the co-expressed genes of FAP in ccRCC in order to truly comprehend its biological characteristics in ccRCC. The heatmap shows the top 25 positively and top 25 negatively linked genes with FAP in ccRCC (Fig. 6A). We used GO and KEGG pathway analysis on the top 50 FAP co-expressed genes in ccRCC to gain a complete understanding of their probable roles as well as molecular mechanisms (Fig. 6B, C). An additional finding from GSEA indicated that the PI3K/AKT pathway was closely related with FAP expression pattern (Fig. 6D). As a consequence of our findings from GO, KEGG, and GSEA, we carried out western blot analysis in ccRCC to examine the impact of FAP on the PI3K/AKT/mTOR signaling cascade. Western blot analysis showed that FAP upregulation elevated the expression profiles of p-PI3K, p-AKT, and p-mTOR without altering those of PI3K, AKT, and mTOR, and that application of BEZ235 attenuated PI3K, AKT, and mTOR phosphorylation (Fig. 7F). Cellular viability was lower in the OE-FAP + BEZ235 group compared to the OE-FAP group (Fig. 7A). The number of apoptotic nuclei was less in the OE-FAP + BEZ235 group compared to the OE-FAP group (Fig. 7B). The migration of ccRCC cell was significantly reduced in the OE-FAP + BEZ235 group compared to the OE-FAP group (Fig. 7C, D). The cell invasion was markedly reduced in the OE-FAP + BEZ235 group compared to the OE-FAP group (Fig. 7E). These results demonstrated that FAP could increase ccRCC carcinogenesis by activating the PI3K/AKT/mTOR signaling pathway.

### Inhibition of FAP reduced the development of ccRCC in vivo

**Fig. 8:**
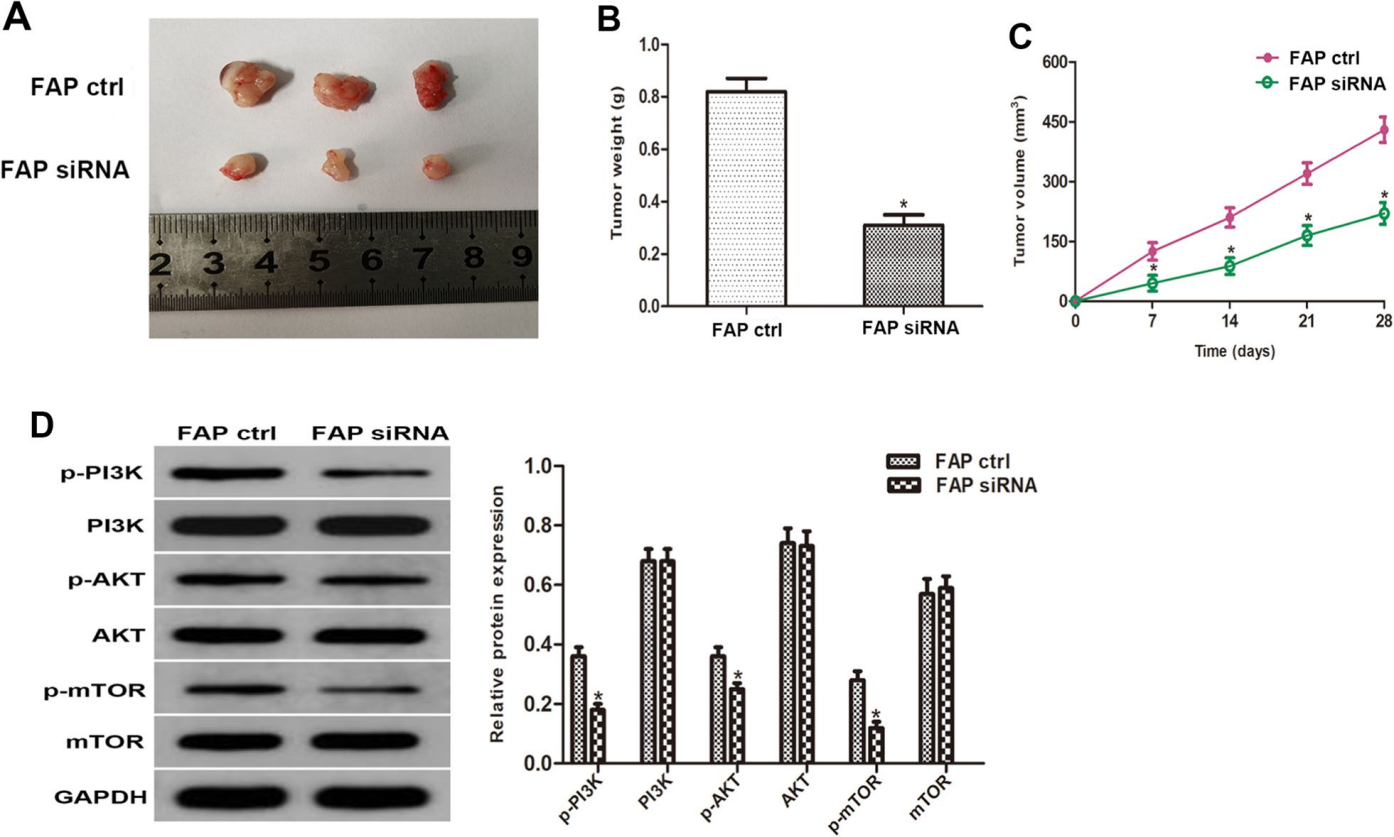
Cancer progression was inhibited by Silencing FAP in an animal model of ccRCC xenografts. **A** The xenograft tumors were excised from the nude mice of each group after sacrifice. **B** The final weight of tumors was analysed. **C** Changes of the tumor volume in each group were monitored every 7 days. **D** The PI3K/AKT/mTOR pathway protein content in xenograft tumor tissues were examined using western blot analysis. *p < 0.05 vs. the Ctrl group

**Fig. 9:**
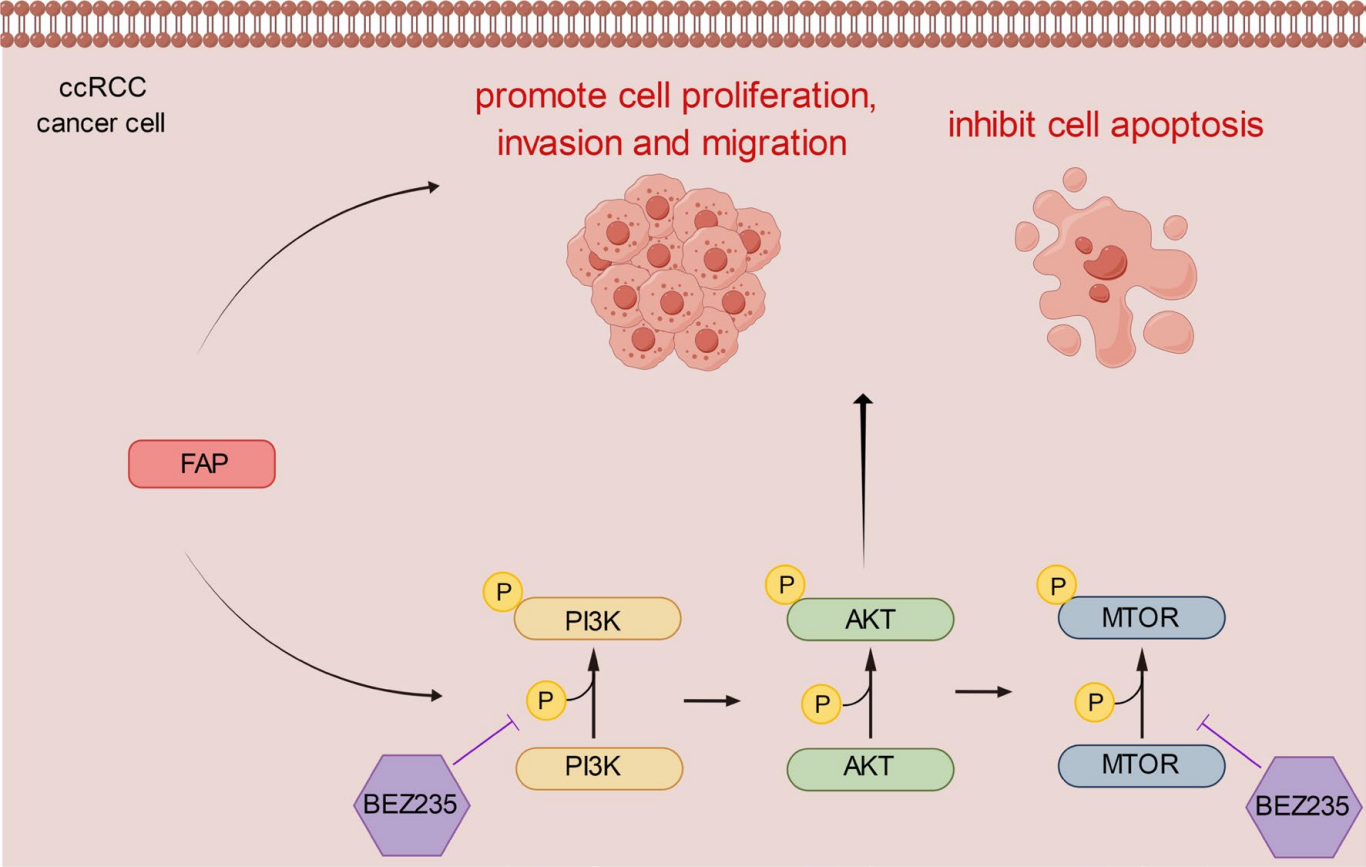
The schematic diagram explains the molecular mechanism underlying the pro-ccRCC effect of FAP. Briefly, FAP modulates the expression of downstream PI3K/AKT/mTOR signaling pathway, thereby promoting ccRCC proliferation, migration and invasion and inhibiting apoptosis

To confirm furthermore the impact of FAP in vivo, a mice ccRCC xenograft models were developed. Subcutaneous injections of Caki-1 cells that had been transfected with si-FAP or si-NC were administered to nude mice. Every week, variations in tumor size in each subgroup were assessed (Fig. 8C). Compared with the control group, xenograft tumor volumes (Fig. 8A) and weights (Fig. 8B) were significantly lower in the si-FAP group after 35 days. Western blot analysis showed that FAP silencing reduced the expression profiles of p-PI3K, p-AKT, and p-mTOR (Fig. 8D). In summary, these results suggested that silencing of FAP suppressed ccRCC development in vivo. Collectively, we have generated a schematic diagram illustrating the progression of FAP promoted ccRCC through the activation of the PI3K/AKT/mTOR signaling pathway, highlighting FAP as a promising therapeutic target for ccRCC (Fig. 9).

## Discussion

RCC is the second most prevalent cancer of the urinary system, particularly ccRCC is the most prevalent and serious pathophysiological subtype, comprising 85–90% of RCC [18]. Due of extensive vasculature, ccRCC can quickly develop and metastasize [19]. 30% of early stage ccRCC patients had metastatic cancer upon detection, and another third will recur following nephrectomy [20]. Despite advancements in health care, ccRCC survivability is low [21]. To enhance the detection and management of advanced stage ccRCC, additional biomarkers must be screened.

CAFs has been recognized as a pro-tumorigenesis role at all stages of tumor progression [22]. FAP, an essential surface marker of CAFs, performs a critical function in tumorigenesis and acts as an immunosuppressant in the cancer microenvironment [23]. There are indications that expression of FAP is significantly up-regulated in tumors with an aggressive nature and a higher proclivity for spreading [24]. Upregulated FAP in breast cancer has been linked to advanced disease, older patients, lymph node metastases, as well as greater mortality rates [25]. A poor prognosis in women with ovarian cancer seems to be directly linked to elevated amounts of FAP expression in their peritoneal or pleural effusions, according to Zhang et al [26]. FAP promoted colorectal cancer angiogenesis by stimulating the ERK and AKT signal transduction pathways [27]. The PTEN/PI3K/AKT and Ras-ERK signaling pathways have been shown to be strongly inhibited in oral squamous cell carcinoma when FAP is suppressed [28]. In ccRCC, meanwhile, the expression level of FAP and its consequences on the disease are still unclear.

An up-regulation of FAP in the ccRCC tissues was detected using data from TCGA, which was verified employing clinical specimens in the current research study. According to the ROC curve's AUC value of 0.708, FAP was a reliable marker for the early detection of ccRCC. FAP was also linked to lymph node metastases and histological subtypes, as indicated by our findings. Moreover, elevated FAP expression in ccRCC patients was strikingly associated with worse OS, PFI, and DSS. According to further nomograms, FAP presented satisfactory performance on predicting clinical outcomes in ccRCC. Together, these findings suggested that FAP may contribute to ccRCC progression. Thus, Caki-1 and ACHN cells were employed to trigger ccRCC progression in our in vitro experiments. As per our findings, ccRCC cell lines, silencing FAP inhibited cellular proliferation, migration patterns, invasion and accelerated cell death, whereas upregulation of FAP had the opposite impact. In vivo research showed suppressing FAP decreased xenograft tumorigenesis. These results are in accordance with the role of FAP in other tumors, including breast cancer, ovarian cancer, colorectal cancer and oral squamous cell carcinoma [25–28].

It is well known that the intracellular signaling pathway PI3K/AKT/mTOR performs a crucial function in carcinogenesis [29]. The PI3K/AKT/mTOR signaling pathway has been shown to be a cell cycle route that is essential for the control of cellular viability, proliferation, migration, as well as differentiation [30]. The PI3K/AKT/mTOR pathway has been found to be abnormally active in renal cell cancer [31]. Co-regulated genes are more likely to influence the fundamental activities and pathways in particular biological contexts [32]. As per our findings via GO, KEGG as well as GSEA investigations, FAP expression is associated with the PI3K/AKT signaling pathway. In order to learn more about the PI3K/AKT/mTOR signaling cascade, we conducted additional experiments. When FAP was knocked down, the PI3K/AKT/mTOR signaling pathway was inhibited in ccRCC cells, whereas FAP upregulation led to the reverse effect. These findings elucidate a novel mechanism by which FAP regulates tumor cells.

## Conclusion

In conclusion, these results demonstrated that FAP significantly stimulates the development of ccRCC tumors via activating the PI3K/AKT/mTOR signaling pathway. Our study has uncovered a novel regulatory mechanism of the PI3K/AKT/mTOR pathway, highlighting FAP as a promising biomarker for both diagnosis and prognosis of ccRCC, as well as a potential therapeutic target in ccRCC.

## Data Availability

The datasets during and analysed during the current study available from the corresponding author on reasonable request.

## Declarations

### Animal studies

Animal care and experimental techniques were done in compliance with the National Institutes of Health Guidelines for the Use of Laboratory Animals. The Animal Care & Use Committee of Wuhan University permitted this study.
